# Evaluating the Volta phase plate for improved tomogram alignment in cryo-electron tomography

**DOI:** 10.1107/S2052252526002575

**Published:** 2026-04-09

**Authors:** Joshua Hutchings, Daniel Ji, Shawn Zheng, Elizabeth A. Montabana, Utz H. Ermel, Ariana Peck, Jonathan Schwartz, Rahel Woldeyes, Mohammadreza Paraan, Mallak Ali, Norbert S. Hill, Hannah Siems, Daniel Serwas, Anchi Cheng, Dari Kimanius, David A. Agard, Clinton S. Potter, Bridget Carragher, Yue Yu

**Affiliations:** a BioHub Redwood City California USA; b Department of Biochemistry and Biophysics University of California, San Francisco San Francisco California USA; Max Planck Institute of Molecular Physiology, Germany

**Keywords:** Volta phase plate, VPP, single-particle analysis, cryo-electron tomography, subtomogram averaging

## Abstract

The Volta phase plate (VPP) can enhance low-frequency phase contrast in cryo-electron microscopy (cryo-EM) and cryo-electron tomography (cryo-ET), but its impact on tomogram alignment has not been systematically assessed. Using sub-2 Å single-particle analysis (SPA) and analytical contrast transfer function (CTF) modeling, we show that despite high-frequency information loss and moderate signal damping, the VPP enhances signal in low-frequency bands that are crucial for tilt-series alignment. We show that VPP tomograms exhibit increased contrast and remain higher in contrast post denoising, for thick and crowded specimens. Quantitative comparison of tomograms acquired with and without the VPP shows improved tilt-axis orientation measurements and a higher fraction of tomograms amenable to local tilt-series alignment. Although acquisition with the VPP yields lower subtomogram averaging (STA) resolution, these results show that the primary benefits of the VPP for cryo-ET are contrast enhancement and improved tilt-series alignment robustness, which are crucial for studying cellular ultrastructure and protein organization in crowded environments, where STA at moderate resolutions remains valuable.

## Abstract 2

We use sub-2 Å single-particle analysis and analytical contrast transfer function modeling to show that the Volta phase plate (VPP) boosts low-frequency signal relevant for cryo-electron tomography (cryo-ET) tilt-series alignment, improving contrast and alignment robustness in thick, crowded samples. Despite reduced subtomogram averaging resolution, these results establish the VPP’s value for cryo-ET in thick, dense specimens, where tilt-series alignment is challenging and moderate-resolution information can still provide valuable biological insights.

## 1. Introduction

Cryo-electron microscopy (cryo-EM) and cryo-electron tomography (cryo-ET) with subtomogram averaging (STA) are essential tools for studying biomolecular structures. In these methods, biomolecules embedded in vitreous ice behave as weak-phase objects and are imaged by transmission electron microscopy (TEM). With conventional wide-beam, bright-field imaging, defocus is deliberately introduced to convert phase contrast into detectable intensity contrast. Using this approach, single-particle analysis (SPA) now routinely reaches near-atomic resolution, with several atomic-resolution examples reported (Yip *et al.*, 2020 ▸; Nakane *et al.*, 2020 ▸; Küçükoğlu *et al.*, 2024 ▸), while cryo-ET with STA has also progressed to resolutions suitable for atomic model fitting, largely driven by advances in image processing software (Tegunov *et al.*, 2021 ▸; Xue *et al.*, 2022 ▸).

Active research continues to advance cryo-EM and cryo-ET, with one major direction focused on improving phase information detection. One computational approach is ptychography-based phase retrieval (Küçükoğlu *et al.*, 2024 ▸), which, despite its widespread success in materials science (Jiang *et al.*, 2018 ▸), remains at an early stage with cryo-EM. A parallel and long-standing effort has been the development of phase plates. Several approaches to phase-plate engineering have been explored, including thin-film-based designs (Danev & Nagayama, 2001 ▸; Danev *et al.*, 2014 ▸), electrostatic phase plates (Schultheiss *et al.*, 2010 ▸) and high-power-density laser-based phase plates. Among these, the laser phase plate (LPP) represents a recent advance and has been shown in principle to produce a stable π/2 phase shift (Schwartz *et al.*, 2019 ▸; Remis *et al.*, 2024 ▸). LPP development remains an active and rapidly advancing area of research, with both continuous (Petrov *et al.*, 2024 ▸; Olshin *et al.*, 2025 ▸) and pulsed (Du & Fitzpatrick, 2023 ▸) configurations under development, and has demonstrated strong potential to provide ideal phase contrast for cryo-EM.

Among available phase-plate designs, thin-film phase plates are comparatively simple to engineer, with the Volta phase plate (Danev *et al.*, 2014 ▸) (VPP) being the most widespread and commercially available example. The VPP uses the electron beam to induce an electrostatic potential on a thin film, generating a phase shift between the scattered and unscattered electrons. Proven in principle in 2014 (Danev *et al.*, 2014 ▸), the VPP was subsequently applied to cryo-EM single-particle analysis using near-zero (Danev & Baumeister, 2016 ▸) and low defocus imaging (Danev *et al.*, 2017 ▸). Comparative studies of imaging with and without the VPP include applications to benchmark proteins (Danev *et al.*, 2017 ▸; Li *et al.*, 2019 ▸), membrane proteins (Danev *et al.*, 2021 ▸) and small complexes (Fan *et al.*, 2019 ▸). Overall these studies showed that the VPP significantly improved image contrast and in some cases facilitated particle alignment, particularly for small particles (Fan *et al.*, 2019 ▸). However, in most applications, VPP datasets did not surpass defocus-based imaging in final resolution. This limitation was later attributed to a spatial-frequency-dependent signal loss introduced by the VPP (Buijsse *et al.*, 2020 ▸), in addition to the previously known constant attenuation from electron scattering in the phase plate material (Danev *et al.*, 2017 ▸).

With continued advances in SPA sample preparation and data processing, and the recognition of VPP-induced signal loss which particularly affects high-resolution signals, VPP-based SPA applications have become less common. Nevertheless, the role of the VPP in cryo-ET remains less well explored. Unlike SPA, where achieving the highest possible resolution is often the primary goal, cryo-ET sometimes focuses on cellular ultrastructure and the spatial organization of protein complexes in crowded environments, where contrast enhancement is highly valuable and moderate-resolution (∼7 Å) subtomogram averages can still be informative. Moreover, accurate alignment of tilt series is fundamental to cryo-ET data interpretation and is one of the major limiting factors in a tomogram’s achievable resolution (Dickerson & Lucas, 2025 ▸). In cryo-ET, dose limitations and additional signal loss in thicker specimens result in substantially lower signal-to-noise per tilt image, making both 2D motion correction and 3D tilt-series alignment challenging. The tilt-series alignment requires not only global alignment of the tilt geometry but also local correction of non-rigid motion and sample deformation. Because the VPP enhances low- to medium-frequency signal, it has the potential to substantially improve tilt-series alignment.

To date, there have been a few systematic benchmarking studies of VPP performance in cryo-ET. In 2017, close-to-focus VPP-based STA of ribosomes demonstrated sub-nanometre resolution without CTF correction, while defocus-based STA achieved similar resolution with CTF correction (Khoshouei *et al.*, 2017 ▸). A later study in 2020 compared defocus-based imaging with close-to-focus and defocused VPP imaging, reporting resolutions in the range ∼4–7 Å where secondary-structure elements are visible (Turoňová, 2020 ▸). In that comparison, close-to-focus VPP produced lower resolution than defocused VPP, which in turn was slightly worse than defocus-based STA. While resolution is an important metric for STA, given the known frequency-dependent signal damping introduced by the VPP, focusing on resolution alone may overlook other potential advantages of the VPP in cryo-ET, particularly those related to tomogram alignment.

Here, motivated by the low- to medium-frequency signal enhancement provided by the VPP and its relevance to tomogram alignment, we present a systematic comparison of VPP and defocus-based (non-VPP) cryo-ET datasets. We examine two types of samples (both unmilled): a relatively thin and sparsely populated phantom (Peck, Yu, Schwartz *et al.*, 2025 ▸) and thick bacterial minicells with densely packed proteins (Breuer *et al.*, 2019 ▸). VPP tomograms were acquired at low defocus (0.7–0.9 µm), while non-VPP tomograms were collected at 2 µm defocus. Each dataset contains ∼100–300 tomograms, enabling statistical analysis of tomogram alignment quality, with a particular focus on thick and crowded specimens. We additionally present sub-2 Å SPA reconstructions of apoferritin acquired with and without a VPP to empirically model VPP-induced signal loss, and we report STA reconstructions from the tomographic datasets to provide a resolution comparison. Together, this work aims to evaluate the impact of the VPP on tomogram alignment and to define practical use cases for VPP imaging in cryo-ET, particularly for specimens that pose challenges for robust alignment.

## 2. Materials and methods

### 2.1. Apoferritin SPA sample preparation

Mouse apoferritin heavy chain (FTH1) was expressed in *Escherichia coli* Lemo21(DE3) (NEB) from a pET24a vector. Fresh transformants were grown overnight in LB and used to inoculate 1 l Super Broth (Teknova). Cultures were grown at 37°C to OD_600_ ∼0.7, induced with 0.5 m*M* IPTG (Sigma-Aldrich), and incubated 16 hr at 18°C before harvesting and freezing. Pellets from 0.5 l culture were resuspended in Buffer A (30 m*M* HEPES pH 7.5, 300 m*M* NaCl, 2 m*M* MgSO_4_) with lysozyme and cOmplete inhibitor (Roche), lysed by sonication, and clarified by centrifugation (13 000 r.p.m., 30 min, 4°C, Ti45 rotor). The supernatant was heated to 70°C for 10 min and centrifuged again. Ammonium sulfate was added to 33.25 g/100 ml; the precipitate was pelleted, resuspended in Buffer B (20 m*M* HEPES pH 7.5, 300 m*M* NaCl), and dialyzed overnight.

The sample was diluted 1:5 and loaded onto a 5 ml HiTrap Q column (Cytiva) equilibrated in Buffer C (20 m*M* HEPES pH 7.5, 75 m*M* NaCl). After washing, FTH1 was eluted with a linear gradient to Buffer D (20 m*M* HEPES pH 7.5, 1 *M* NaCl). Peak fractions were analyzed by SDS–PAGE and concentrated using 100 kDa MWCO Amicon filters (MilliporeSigma). Size-exclusion chromatography was performed on a Superdex 200 16/60 column (Cytiva) using SEC buffer (20 m*M* HEPES pH 7.5, 150 m*M* NaCl) at 0.35 ml min^−1^. Peak fractions were pooled, concentrated to ∼19 mg ml^−1^ (BCA assay; Thermo Fisher Scientific), and flash-frozen without cryoprotectant.

Quantifoil Cu 200 mesh 1.2/1.3 grids were glow-discharged with a PELCO easiGlow. Plunge freezing was done with a Thermo Fisher Scientific (TFS) Vitrobot set at 10°C and 95% humidity.

### 2.2. Sample preparation for two-protein phantom

The two-protein phantom was generated from a mixture of cell lysates and purified proteins using a protocol similar to that described previously for a realistic cryo-ET phantom dataset (Peck, Yu, Schwartz *et al.*, 2025 ▸). In particular, the cell lysates were prepared from a HEK293T line with a C-terminal GFP knock-in on TMEM192, provided by M. Leonetti’s group at the Biohub, San Francisco (Cho *et al.*, 2022 ▸). Cells were lysed in a hypotonic homogenization buffer (25 m*M* Tris-HCl pH 7.5, 50 m*M* sucrose, 0.2 m*M* EGTA, 0.5 m*M* MgCl_2_) using a 23G syringe to generate shearing forces (Hein *et al.*, 2025 ▸). The lysate was then mixed with sucrose buffer (2.5 *M* sucrose, 0.2 m*M* EGTA, 0.5 m*M* MgCl_2_). The nuclear fraction was removed by centrifugation at 1000*g* for 10 min. Lysosomes bearing the TMEM192-GFP fusion were subsequently captured onto electron-microscopy grids functionalized with GFP nanobodies (Wang, Liu *et al.*, 2020 ▸; Wang, Yu *et al.*, 2020 ▸). This technique was adapted for organelle isolation in collaboration with David Agard’s group at the University of California, San Francisco.

Plunge freezing was performed using a Leica GP2 set to 4°C and 95% humidity. The lysate was pipetted up and down (20 strokes) in 10 µl volumes and applied for over ten repeated rounds, resulting in an overall application of 100 µl lysate to the grid surface. The grid was then washed twice with PBS by pipetting. Before adding the purified protein, the excess buffer remaining on the grid was removed. PP7 virus-like particles (2.1 mg ml^−1^) were then added sequentially in 4–6 µl aliquots. The grid was back-side blotted for 6 s and plunge-frozen in liquid ethane.

### 2.3. Sample preparation for *Mycoplasma mycoides* JCVI-Syn3A

JCVI-Syn3A cells were obtained from John Glass’s lab at J. Craig Venter Institute (Breuer *et al.*, 2019 ▸). Cells were thawed from −80°C and gently mixed until ready for transfer into culture. Culture tubes containing 1 ml SP4 medium were prepared, and approximately 10 µl of thawed material (or a small scraped portion of the frozen stock) was added to each tube. A separate uninoculated tube of media was maintained to monitor pH changes, which typically occurred within 1–3 days. For scale-up, cultures were diluted 1:100 into fresh pre-warmed medium and grown overnight. Quantifoil Cu 200 mesh 2/1 grids were glow-discharged using a PELCO easiGlow. Grids were plunge-frozen using a Leica GP2 set to 4°C and 95% humidity. A 4 µl aliquot of cell pellet was applied to each grid, blotted for 6 s, and plunge-frozen in liquid ethane.

### 2.4. Apoferritin SPA data collection

For both datasets, VPP and non-VPP, a Titan Krios G4 microscope (Thermo Fisher Scientific, TFS) with a *C*_s_ of 2.7 mm and an XFEG was used. Images were acquired at 300 keV, 10 eV slit using a Selectris X energy filter and a Falcon 4i detector. The total dose was 45 e Å^−2^ and the pixel size was 0.288 Å in super-res mode. For the non-VPP datasets, targeted defoci are centered about 1 µm. Aberration-free image shift (AFIS) data were acquired with TFS *EPU* with the ‘Faster’ setting in the ‘Acquisition Mode’. For the VPP datasets, targeted defoci are centered about 0.6 µm and AFIS acquisition was in the ‘Accurate’ setting. Three hundred exposures were acquired per VPP spot with ∼20 minutes charging using the exposure setting. The charging time was determined by *Sherpa* with a targeted phase shift of 0.35π.

### 2.5. Apoferritin SPA data analysis

Apoferritin SPA data were processed in *cryoSPARC* (Punjani *et al.*, 2017 ▸). Forty movie frames were rendered from EER frames. Templates generated from EMDB-11668 were utilized for particle picking. Particles were cleaned through a round of 2D classification, with a 30 Å high-pass filter applied to VPP datasets to improve 2D classification quality. For both datasets, 300 702 particles were used downstream, including *ab initio* model generation and subsequent homogeneous refinement. Homogeneous refinement was applied with octahedral symmetry. Refinement included global CTF refinement and per-particle defocus refinement. The global CTF refinement incorporated spherical aberration, tetrafoil and anisotropic magnification. No per-micrograph or per-particle phase-shift refinement was performed. The final reconstruction and FSC curves were validated within *cryoSPARC*.

### 2.6. CTF modeling and CTF density calculation

The CTF was modeled as a phase-contrast transfer function without amplitude contrast. Aberrations include defocus and spherical aberration (*C*_s_ = 2.7 mm) at 300 kV. The CTF was evaluated on a two-dimensional reciprocal-space grid and convolved with an isotropic envelope function, 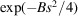. For the VPP, a cut-on frequency of 0.004 Å^−1^ was applied, below which the phase shift is 0°. The cut-on frequency *k*_*h*_ was estimated by *k*_*h*_ = *r*_*h*_/*f*λ [equation (3) in Danev & Nagayama (2001 ▸)] where *r*_*h*_ is the effective VPP phase-shift patch radius, *f* the effective focal length (3.5 mm) and λ the electron wavelength (1.97 pm). Based on a previously reported phase-shift patch size to be ∼11× larger than the beam size in the back focal plane (Danev *et al.*, 2014 ▸), we estimate *r*_*h*_ to be ∼275 nm. No cut-off frequency is included. Signal attenuation due to the VPP was estimated from the difference in Rosenthal–Henderson *B* factors measured in apoferritin datasets (67.6 Å^2^ without VPP and 100.8 Å^2^ with VPP), together with an independently measured ∼12% reduction in beam intensity without a specimen. The *B* factors represent cumulative effects from protein structure, imaging and processing. Because the same sample preparation and processing pipeline were used in both conditions, the *B*-factor difference primarily reflects the effect of the VPP. Finally, the CTF density within each predefined spatial-frequency range (R1–R4) was calculated as the summed |CTF| values across the corresponding frequency bins and normalized by the number of bins in that range.

### 2.7. Cryo-ET data collection

For both the minicell and two-protein phantom, tilt series were acquired on a Titan Krios G4 microscope (Thermo Fisher Scientific, TFS) with an XFEG and a *C*_s_ of 2.7 mm, operated at 300 keV. A 10 eV energy filter slit with a Selectris X and a Falcon 4i detector were used. Tilt series were collected with TFS *Tomography 5* over a tilt range of ±45° in 3° steps with dose-symmetric collection schema. The total dose over vacuum was kept at about 120 e Å^−2^, and images were recorded at a pixel size of 1.51 Å (non-super-res). For non-VPP datasets, the nominal defocus targets are centered around 2 µm; on average 6–7 parallel image-shifted acquisitions were taken per stage position. For VPP datasets, the target defocus was 0.7–0.9 µm, and one tilt series was acquired per stage position. For the VPP minicell, each VPP spot was charged using the acquisition imaging condition for ∼23 min before acquisition, and 30 tilt series were acquired at each VPP position.

### 2.8. Cryo-ET preprocessing and alignment comparison

Beam-induced motion correction, tilt-series alignment and tomogram reconstruction were performed using *AreTomo3* v2.2.2 (Peck, Yu, Paraan *et al.*, 2025 ▸). Every ten EER frames were summed to generate rendered frames, which were used to measure and correct both global and local beam-induced motion, with the local motion measured on 5 × 5 patches. Local CTF parameters were estimated for each tilt image on overlapping patches. For VPP tomograms, the phase-shift search option was turned on and the search range was 35–105°. For the two-protein phantom dataset, tilt-series alignment was performed on the CTF-corrected tilt series with global alignment followed by 4 × 4 patch-based local alignment. The tilt-axis orientation was automatically determined from the alignments. For both the two-protein phantom and minicell, and both with and without VPP, tomograms were denoised using *DenoisET* (Peck, Yu, Paraan *et al.*, 2025 ▸) with the same denoising model. To ensure comparable post-specimen dose ranges between VPP and non-VPP datasets, non-VPP tomograms were filtered using the minimum and maximum post-specimen doses from the VPP dataset, divided by a damping factor of 0.88 (measured by beam intensity drop with no specimen). Non-VPP tomograms were retained only if their zero-tilt post-specimen dose fell within this minimum and maximum post-specimen dose range.

### 2.9. Particle picking

Particle picking in all four structures was performed using a combination of manual annotation, deep-learning-based automated detection and 2D minislab classification. The picking processing for each structure is described below.

For PP7 virus-like particles (VLPs) in the non-VPP two-protein phantom datasets, particles were manually identified in 14 denoised tomograms using *ArtiaX* (Ermel *et al.*, 2022 ▸) and *ChimeraX* (Meng *et al.*, 2023 ▸) and exported in *copick* format (Harrington *et al.*, 2024 ▸). A total of 22 manually picked particles were used to train *Octopi* (see *Data Availability*), a modular deep-learning framework for particle picking. The trained model was then applied to all 197 tomograms, yielding 490 automatically detected VLPs. These particles were further filtered using the minislab 2D classification method described previously (Peck, Yu, Schwartz *et al.*, 2025 ▸) (see *Data Availability*), in which 3D subtomograms are projected into 2D minislabs and subjected to 2D classification using *RELION* (Burt *et al.*, 2024 ▸), resulting in 290 particles retained. VPP VLPs were picked using the same workflow. Twenty-five particles were manually picked from a single denoised tomogram to train *Octopi*, and the resulting model was applied to 115 tomograms, yielding 514 automatically detected VLPs. After minislab 2D classification, 323 particles were retained.

For 80S ribosomes in the non-VPP two-protein phantom datasets, particle picking was performed on all 197 tomograms. A total of 225 particles were manually identified from four denoised tomograms and used to train *Octopi*, which was then applied to the full dataset, yielding 15 643 automatically detected particles. After minislab 2D classification, 11 479 particles were retained. For the corresponding VPP dataset, particle picking was performed on all 115 tomograms. A total of 159 particles were manually identified from three denoised tomograms and used to train *Octopi*, yielding 8996 automatically detected particles. After minislab 2D classification, 3710 particles were retained. Tomogram and particle counts are summarized in Table 1 ▸.

For VPP 70S ribosomes from minicells, 5840 particles were manually identified from six denoised tomograms [CryoET Data Portal (https://cryoetdataportal.czscience.com) DS-10452-RN-18181, 18182, 18187, 18188, 18194 and 18268]. The manually picked ribosomes were used to train *Octopi*, and the resulting model was applied to a manually selected subset of 29 tomograms from the full dataset of 107 tomograms. To improve automated particle detection, membrane segmentation was performed using *MemBrain-seg *(Lamm *et al.*, 2024 ▸) and used as a mask to exclude false positives on the membrane or outside of the minicells, yielding 18 203 particles as input for STA. For 70S ribosomes from minicells acquired without the VPP, a total of 2041 particles were manually picked from three denoised tomograms and used to train *Octopi*, which was then applied to 39 manually selected tomograms. Subsequent cleaning with the membrane segmentation mask yielded 38 823 particles for STA.

### 2.10. Subtomogram averaging

For each species, subtomogram averaging (STA) for both non-VPP and VPP datasets was performed using *RELION-5* (Burt *et al.*, 2024 ▸) and *py2rely*, a software package integrating *CCP-EM Pipeliner* job-execution utilities for *RELION* (see *Data Availability*). Coordinates from *Octopi* and 2D minislab classification were imported into *RELION-5* using utilities from *py2rely*. The following resampled low-pass filtered (LPF) references and pixel sizes were used for initial reference-based refinements using *py2rely*: VLPs – 40 Å LPF, EMDB-41917, bin6, 9.06 Å pixel^−1^; 80S ribosome – 40 Å LPF, EMDB-3883, bin6, 9.06 Å pixel^−1^; and 70S ribosome – 50 Å LPF, EMDB-10677, bin4, 6.04 Å pixel^−1^. For fair comparison of non-VPP and VPP, particle counts were equalized for each species based on the size of the smaller dataset by selecting a random subset of the larger dataset following these binned initial alignments. Nevertheless, in each case the larger dataset was also processed to demonstrate the capabilities of the refinement pipeline (Table 2 ▸, Supplementary Fig. 10). For 70S ribosomes, an additional round of 3D classification without alignment was performed at bin4 (6.04 Å pixel^−1^) using five classes without a tau fudge factor. In both cases this yielded one class of high-quality particles, which formed the basis for choosing an equal number of input particles for high-resolution refinements, described below.

All datasets were refined at bin2 (3.02 Å pixel^−1^) followed by unbinned (1.51 Å pixel^−1^) high-resolution tomography refinements (Burt *et al.*, 2024 ▸). Two cycles of refinements were performed, comprising the following steps in *RELION-5*: reconstruction, postprocessing, CTF refinement, Bayesian polishing, extraction, reconstruction, 3D refinement and postprocessing. To isolate the benefit of each refinement step in VPP and non-VPP datasets, CTF refinement or Bayesian polishing were omitted from the pipeline, keeping all other steps the same (Supplementary Fig. 11).

Bin2 and unbinned particle-shaped masks for high-resolution refinements were obtained from an initial *py2rely* run of the non-VPP datasets for each species. Briefly, preliminary reconstructions at bin2 and unbinned pixel sizes were low-pass filtered to a resolution determined by Fourier shell correlation (FSC) at the 0.5 threshold of the unmasked half-maps, using the false-discovery-rate (FDR)-based FSC utility (*FDR_FSCcontrol.py*) (Beckers *et al.*, 2019 ▸). A 10-pixel soft edge was applied using *relion_image_handler*. Final resolution estimates were reported using the same unbinned particle-shaped masks as refinements, except for VLPs, which used a soft-edge mask obtained from the asymmetric subunit. Particle counts and resolution estimates are summarized in Table 2 ▸. Note that final particle counts are not equal between species, as a small fraction of particles at the edge of tomograms cannot be extracted after rounds of refinement and extraction. Rosenthal–Henderson *B*-factor plots were calculated from subsets of reconstructed particles and their half-map FSCs, using subsets with increasing size in 10% increments.

## 3. Results

### 3.1. Sub-2Å apoferritin single particle analysis (SPA) with and without the Volta phase plate (VPP)

**Figure 1:**
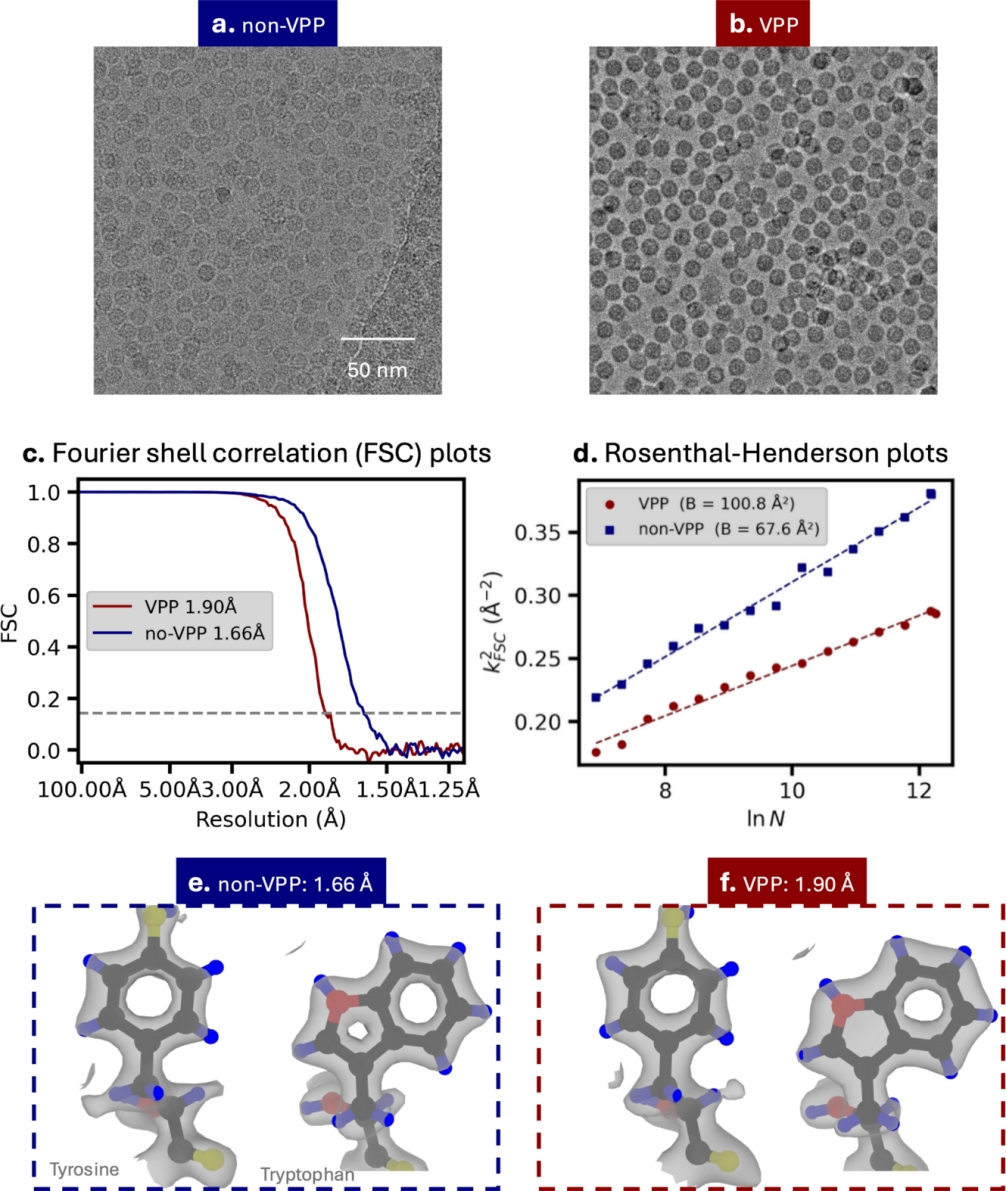
Comparison of apoferritin single-particle analysis (SPA) with and without the Volta phase plate (VPP). (*a*, *b*) Representative micrographs collected without (defocus: 1.31 µm) and with the VPP (defocus: 0.64 µm; phase shift: 70.1°) illustrating the increased low-frequency contrast introduced by the phase plate. (*c*) Fourier shell correlation (FSC) curves for non-VPP and VPP reconstructions (1.66 Å for non-VPP and 1.90 Å for VPP at the gold-standard FSC = 0.143 criterion). (*d*) Rosenthal–Henderson plots for non-VPP and VPP datasets (fitted *B* factors: 67.6 Å^2^ for non-VPP and 100.8 Å^2^ for VPP), where *K*_FSC_ is the inverse of resolution obtained with *N* particles. (*e*, *f*) Apoferritin reconstructions obtained without (1.66 Å) and with the VPP (1.90 Å), shown with docked atomic models highlighting tyrosine and tryptophan side chains.

To assess the impact of VPP-induced signal damping especially at high resolutions, we collected apoferritin SPA datasets using identical sample preparation and processed them using the same refinement pipeline. The VPP dataset was acquired at lower defocus, yet the two datasets showed comparable CTF fit quality (Supplementary Fig. 1). Representative micrographs [Fig. 1 ▸(*a*, *b*)], acquired at defocus values of 0.95 µm (non-VPP) and 0.64 µm with a phase shift of 70.1° (VPP), illustrate the increased contrast introduced by the VPP. To ensure representative phase-plate processing, only micrographs with phase shifts greater than 30° were used.

2D classification yielded a similar good-particle (with class averages better than 4 Å) percentage: the non-VPP dataset had 73% particles in good 2D classes, while the VPP dataset increased from 38% to 71% when a 30 Å high-pass filter (HPF) was applied (Supplementary Fig. 2). The HPF’s effect on the non-VPP data is negligible and seems to be VPP-data-specific, consistent with the previous apoferritin study using the VPP (Li *et al.*, 2019 ▸). This effect likely arises when particles have some very similar projections in low resolution; with VPP-enhanced contrast, alignment can be biased toward dominant low-frequency features, resulting in more mis­aligned classes. An HPF mitigates this effect, with a 30 Å cut-off empirically performing best among tested values.

With the same number of particles (300 702), the non-VPP dataset reached a resolution of 1.66 Å, whereas the VPP dataset reached 1.90 Å [Fig. 1 ▸(*c*)]. *B* factors derived from Rosenthal–Henderson (Rosenthal & Henderson, 2003 ▸) plots yielded values of 67.6 Å^2^ for the non-VPP and 100.8 Å^2^ for the VPP reconstructions [Fig. 1 ▸(*d*)], providing a measure of VPP-induced damping of coherent high-resolution signals. Nevertheless, both reconstructions exhibit clear near-atomic features [Fig. 1 ▸(*e*, *f*)]. Table 3 ▸ summarizes the acquisition and processing statistics.

Despite the signal attenuation, the VPP dataset still produced a 1.9 Å reconstruction with the same particle count and comparable number of micrographs, indicating that VPP imaging remains compatible with near-atomic-resolution SPA. Because both datasets share identical preparation and processing, and the measured ice thickness overall is comparable (Supplementary Fig. 3), the difference between their fitted *B* factors provides an empirical estimate of VPP-induced signal attenuation and is used in the following section to inform our analysis.

### 3.2. Analytical CTF signal across defocus and phase-shift conditions

**Figure 2:**
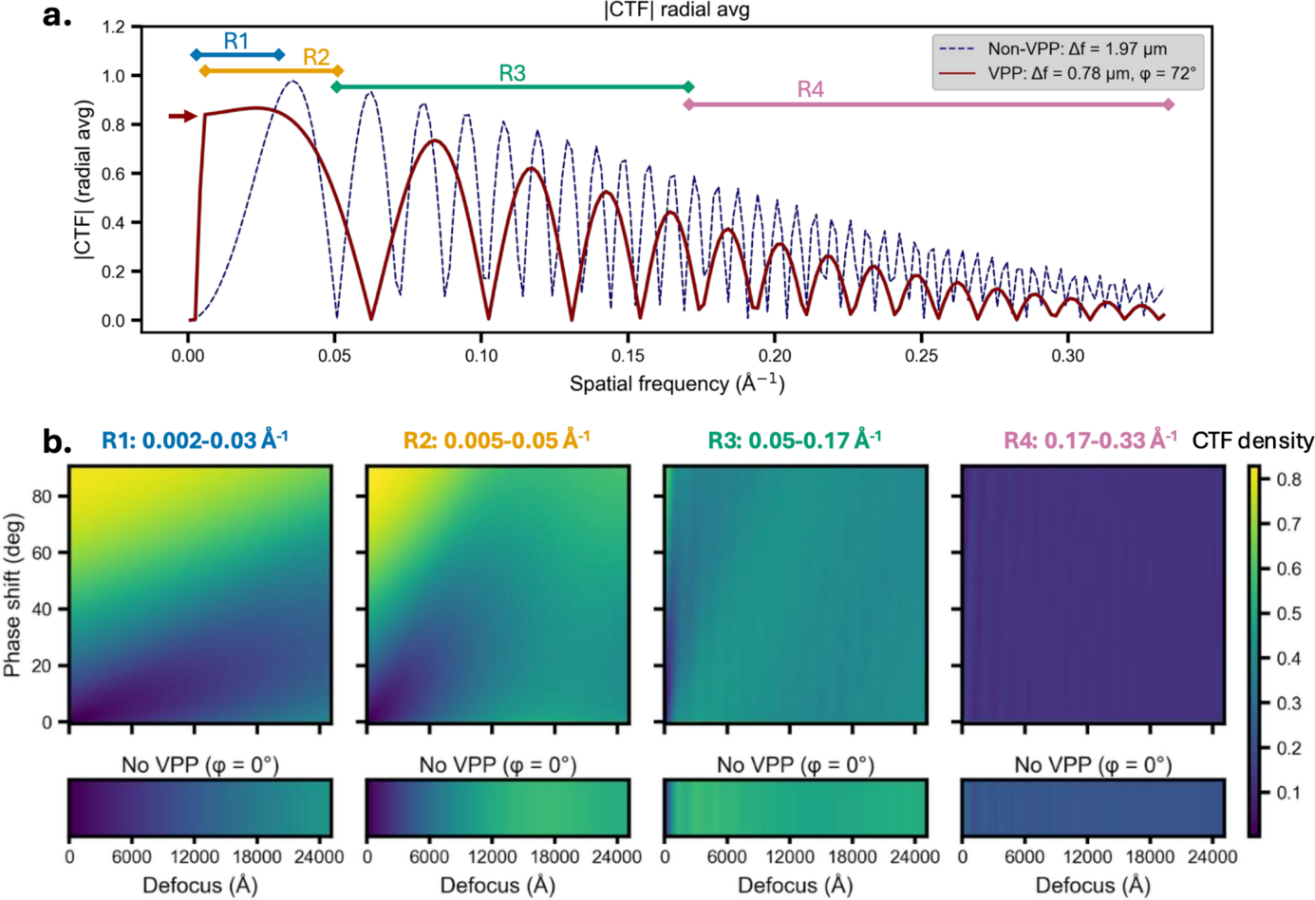
Analytical |CTF| density (integrated absolute value of the contrast transfer function) with and without incorporating VPP-induced phase shift and signal attenuation. (*a*) Radial averages of |CTF| comparing non-VPP imaging (dashed blue; Δ*f* = 1.97 µm) and VPP imaging (solid red; Δ*f* = 0.78 µm, phase shift φ = 72°), including VPP cut-on frequency (red arrow) and VPP-associated signal damping. Δ*f* and φ are median values from the minicell cryo-ET datasets used in this study. Colored segments indicate the four spatial-frequency ranges used for integration: R1 (0.002–0.03 Å^−1^), R2 (0.005–0.05 Å^−1^), R3 (0.05–0.17 Å^−1^) and R4 (0.17–0.33 Å^−1^). (*b*) Heatmaps of integrated |CTF| values computed over a grid of defocus (0–2.5 µm) and phase-shift (0–90°) conditions for each spatial-frequency range (R1–R4). The top row shows calculations including VPP-induced attenuation, while the bottom row is without VPP attenuation (and φ = 0°). Phase shift is plotted along the vertical axis and defocus along the horizontal axis. The color scale indicates normalized |CTF| density.

Using the SPA results as an empirical measure of VPP-induced coherent signal damping, we constructed an approximate CTF model for the VPP. It incorporates beam intensity drop due to VPP scattering, an estimated phase-shift cut-on frequency, and high-resolution signal attenuation inferred from *B*-factor differences [Fig. 2 ▸(*a*)]. We used this model to explore how the VPP alters signal across spatial-frequency ranges relevant to cryo-ET. Specifically, we analyzed four frequency bands [R1–R4; Fig. 2 ▸(*a*)], quantifying the signal by integrating |CTF| values over a grid of defocus and phase-shift conditions [Fig. 2 ▸(*b*)].

The lowest-frequency band, R1 (0.002–0.03 Å^−1^), covers the spatial-frequency range most critical for local motion correction and patch-based tilt-series alignment. The lower bound of 0.002 Å^−1^ reflects typical alignment patch sizes, usually 1/8 to 1/4 of the field of view, while the upper bound of 0.03 Å^−1^ is set by the frequency at which the spectral signal-to-noise ratio (Baxter *et al.*, 2009 ▸) (SSNR) > 1. The upper bound also on the bulk mark agrees with the high-frequency cut-off in a recent study where large *B* factors (1500–3000 Å^2^) were used to assist motion correction (Kong *et al.*, 2025 ▸). Within this band, the |CTF| density increases with phase shift [Fig. 2 ▸(*b*), R1 top], showing a pronounced enhancement relative to non-VPP imaging for phase shifts above ∼45° and defocus below ∼1.5–2 µm, and reaching a maximum at zero defocus and a 90° phase shift. In contrast, without the VPP the signal increases monotonically with defocus [Fig. 2 ▸(*b*), R1 bottom], which explains the conventional use of high defocus values in cryo-ET. The enhancement signal of R1 by the VPP is particularly relevant for improving alignment performance.

The next frequency range is R2 (0.005–0.05 Å^−1^), where the lower bound reflects the size of large particles that can be reliably identified and aligned in cryo-ET, while the upper bound is determined by the tomogram voxel size and the largest binning factors used during initial 3D classification. In this range, the |CTF| density is enhanced under VPP conditions at low defocus (< ∼7500 Å) and high phase shifts (> ∼60°). For both R1 and R2, a practical range of accessible defocus and phase-shift combinations exists where the VPP is expected to provide a benefit. The experimental conditions used for cryo-ET data in this study fall within this favorable region, with a median defocus of ∼7800 Å and a median phase shift of ∼72°, although the phase shift inevitably evolves during data acquisition as the VPP charges.

R3 (0.05–0.17 Å^−1^) corresponds to frequencies relevant for intermediate alignment refinement and was defined based on the intermediate binning factors (bin 4 and bin 2) used in subtomogram averaging in this study. In this range, |CTF| density exhibits [Fig. 2(*b*), R3 top] slightly damped signals with VPP compared to non-VPP (bottom). They show comparable signals only at very low defocus (< ∼1000 Å) combined with high phase shifts (> ∼60°). Although setting such a low defocus is possible, albeit with a possibly low success rate, CTF estimation may become increasingly unreliable. As a result, we did not acquire data in this defocus range. The final range R4 (0.17–0.33 Å^−1^) represents the high-frequency regime where the overall |CTF| density is reduced under VPP conditions, reflecting the impact of high-frequency signal damping.

In R3 and R4, the CTF density exhibits an oscillatory dependence on defocus at low defocus, appearing as mostly vertical ‘stripes’. This is likely because of the wide continuous band of signal between successive CTF zero crossings at low defocus. As defocus varies, the zero crossings shift and result in observable local extrema in the band-integrated signal. With increasing defocus, the CTF oscillates more rapidly with spatial frequency and the local extrema in the CTF density becomes less distinct (Supplementary Fig. 4).

Taken together, these calculations show that the VPP’s potential benefits in cryo-ET are frequency-dependent: it enhances low-frequency bands essential for tilt-series alignment across an experimentally accessible range of defocus and phase shifts, while its attenuation of high-frequency components explains the worse STA resolutions observed with VPP data. Practically, only R1 and R2 increase the integrated signal in a robust and readily achievable regime both for operation and data analysis. In contrast, the conditions required to extract phase-plate benefits in R3 are very challenging and the VPP is not expected to enhance signal in R4. Therefore, tilt series were acquired with defocus values that lie within the favorable R1–R2 region, and we assess the impact of the VPP on tilt-series alignment and subtomogram averaging.

### 3.3. Visual comparison of VPP and non-VPP tomograms

**Figure 3:**
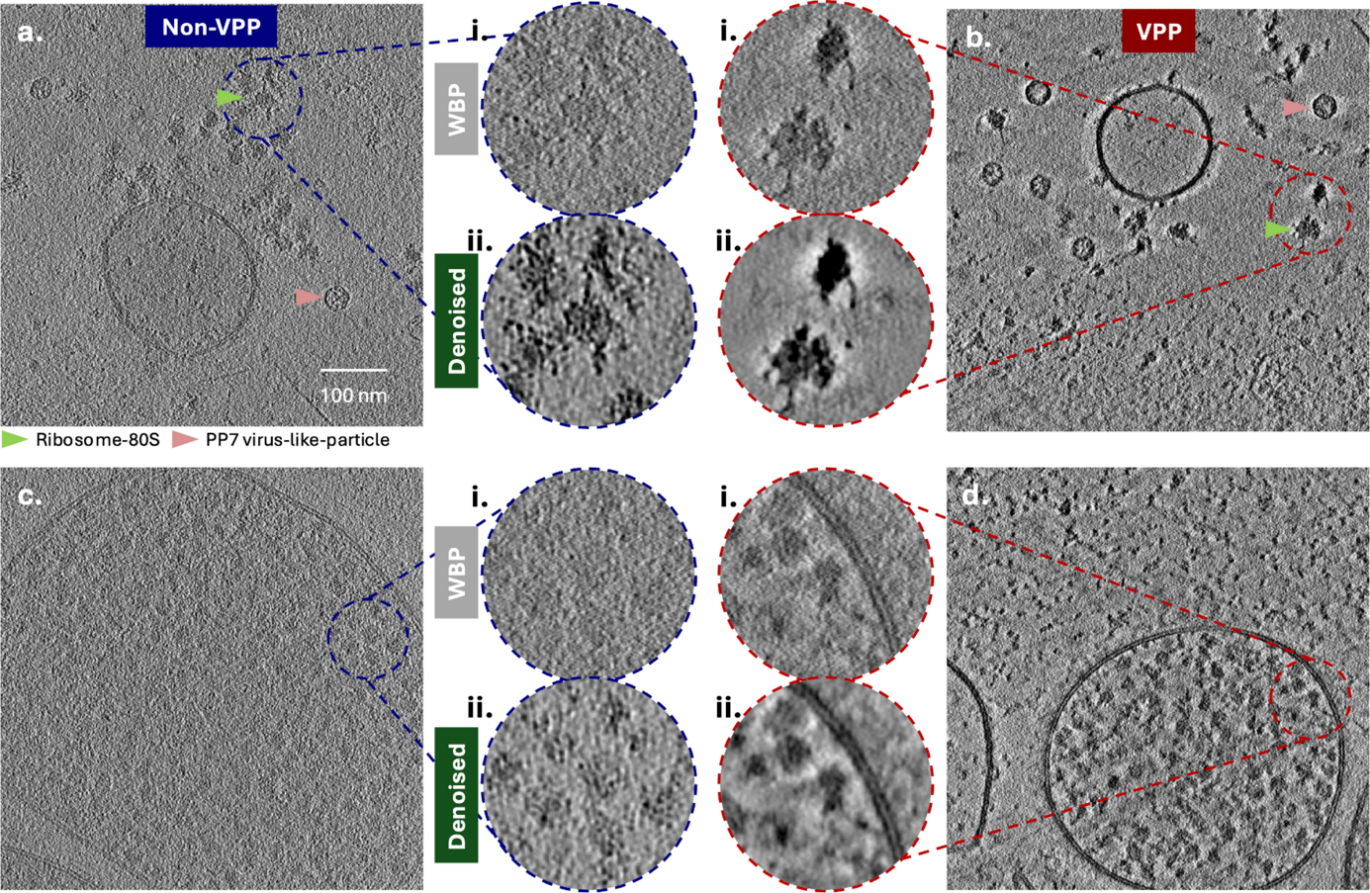
Visualization of tomograms acquired with and without a Volta phase plate (VPP): two-protein phantom and near-minimal cells (minicells) *Mycoplasma mycoides* JCVI-Syn3A. (*a*, *b*) 2 nm slabs from the two-protein phantom recorded without VPP [(*a*), 195 nm thickness measured by *AreTomo3*] and with VPP [(*b*), 165 nm thickness]. Magnified insets highlight selected regions reconstructed with weighted back-projection (*a*1, *b*1) and after denoising (*a*2,*b*2). The green arrow indicates example 80S ribosomes, and the pink arrow marks PP7 virus-like particles. (*c*, *d*) Tomograms of bacterial minicells acquired without VPP [(*c*), 322 nm thickness] and with VPP [(*d*), 320 nm thickness]. Magnified insets show regions containing 70S ribosomes, presented both before and after denoising.

To evaluate the VPP-introduced effect on cryo-ET, we acquired paired VPP and non-VPP datasets from two specimen types: a two-protein phantom sample (Peck, Yu, Schwartz *et al.*, 2025 ▸) and near-minimal *Mycoplasma mycoides* JCVI-Syn3A (Breuer *et al.*, 2019 ▸) (minicells). Across both samples, VPP tomograms exhibit a clear enhancement in image contrast. This effect is particularly evident for the minicell tomograms, which have a median thickness of ∼262 nm, where contrast remains better than non-VPP post denoising. The sample thickness reported is measured by *AreTomo3* (Peck, Yu, Paraan *et al.*, 2025 ▸), based on specimen boundaries identified in binned reconstructions. For the relatively thin and sparsely populated two-protein phantom, we compared tomograms acquired without VPP [Fig. 3 ▸(*a*); defocus: 1.85 µm; thickness: 195 nm] and with VPP [Fig. 3 ▸(*b*); defocus: 0.87 µm; thickness: 165 nm; phase shift: 81°]. The VPP tomogram exhibits visibly higher contrast, including clearer depictions of 80S ribosomes (green arrow) and PP7 virus-like particles (VLPs) (pink arrow), a difference that is evident in the weighted back-projection reconstructions [Fig. 3 ▸(*a*)i, (*b*)i]. After denoising [Fig. 3 ▸(*a*)ii, (*b*)ii], the non-VPP tomogram presents a similar level of structural detail to the VPP tomogram. An additional non-VPP example that is thinner and has higher defocus is shown in Supplementary Fig. 5. The contrast varies with defocus and thickness, but the observed trend that VPP tomograms have more contrast before denoising persists. For thicker and more crowded samples, such as intact bacterial minicells, the contrast benefit of VPP imaging is even more pronounced. The non-VPP minicell tomogram [Fig. 3 ▸(*c*); 1.96 µm defocus, 322 nm thickness] shows limited recognizable structure prior to denoising, making visual identification of 70S ribosomes challenging. In contrast, the corresponding VPP tomogram [Fig. 3 ▸(*d*); 0.86 µm defocus, 320 nm thickness] reveals substantially more details both before and after denoising. All four datasets were denoised using the same general *DenoisET* model (Peck, Yu, Paraan *et al.*, 2025 ▸).

### 3.4. Alignment improvement of VPP tomograms of minicells

**Figure 4:**
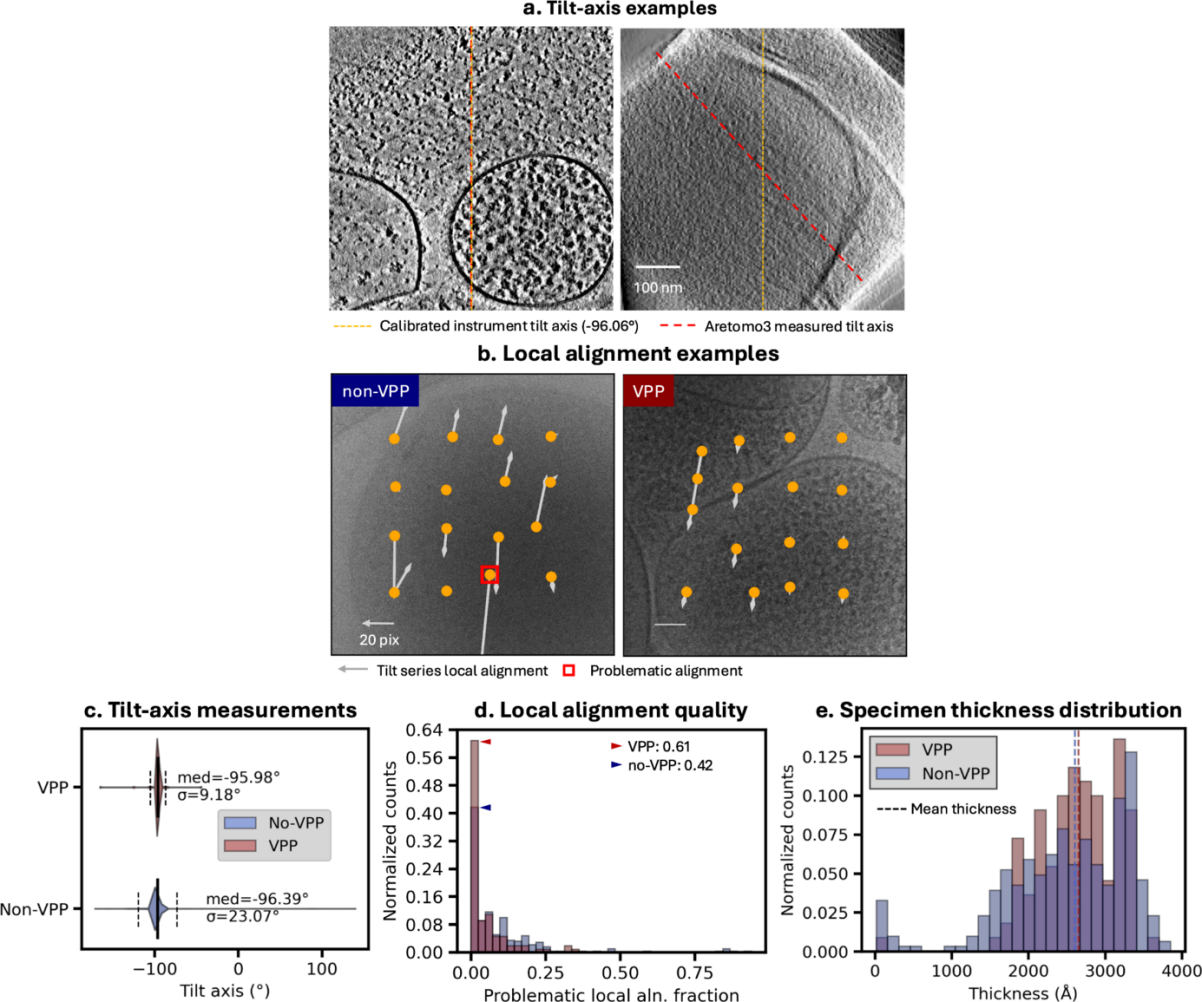
Tomogram alignment quality assessed by *AreTomo3* for minicells of *Mycoplasma mycoides* JCVI-Syn3A acquired with and without a Volta phase plate (VPP). (*a*) Example tomographic slices illustrating *AreTomo3*-measured tilt-axis orientation. In the left example, the measured tilt axis (red) closely matches the calibrated instrument tilt axis (yellow, −96.06°). In the right example, the measured axis deviates substantially from the calibrated value, indicating poor global alignment. (*b*) Tilt-series images non-VPP (left) and VPP (right), annotated with *AreTomo3*-measured local shifts. The non-VPP example contains a problematic local shift flagged by *AreTomo3* (red box), whereas the VPP example shows no such issues. (*c*) Distributions of measured tilt-axis orientations for VPP and non-VPP tomograms. The VPP dataset exhibits a smaller standard deviation, indicating more consistent tilt-axis determination. (*d*) Distributions of problematic local alignment fractions for VPP and non-VPP datasets. A larger fraction of VPP tomograms (61%) show zero problematic local shifts, compared with 42% for non-VPP tomograms. (*e*) Comparison of *AreTomo3*-measured thickness for VPP and non-VPP datasets, which indicates that the specimen-thickness distributions are comparable between the two datasets.

To evaluate whether the low-frequency benefits predicted by the analytical CTF calculations translate into improved tomogram alignment, we examined the alignment metrics for VPP and non-VPP datasets generated by *AreTomo3* (Peck, Yu, Paraan *et al.*, 2025 ▸). *AreTomo3* performs fiducial-free tilt-series alignment, during which the tilt-axis orientation is determined, and the tilt series is aligned in 3D with patch-based local corrections to account for non-rigid motion and distortion. The accuracy of the tilt-axis estimate serves as a measure of global alignment quality, with representative examples shown in Fig. 4 ▸(*a*); large deviations from the instrument-calibrated orientation [Fig. 4 ▸(*a*), right] always indicate poor global alignment. Another alignment metric is derived from local alignments; *AreTomo3* flags problematic local alignments when the measured values deviate from a continuous spatial distribution across the tilt series. Two examples using 4 × 4 patches are shown [Fig. 4 ▸(*b*)], with one exhibiting a problematic local alignment (red box, left) and the other showing continuously varying local alignments across neighboring patches (right).

As discussed in the previous section, VPP tomograms in this study were acquired at defocus values pushed as low as possible while still allowing robust CTF estimation. Accordingly, VPP minicell tomograms have a median defocus of 0.78 µm compared with 1.97 µm for non-VPP datasets [Supplementary Fig. 6(*a*)]. Despite this difference, the estimated CTF fit quality differed modestly, with a slightly worse median CTF resolution for the VPP dataset [8.48 Å versus 7.7 Å; Supplementary Fig. 6(*b*)]. Phase-shift measurements show the expected evolution during acquisition, with a median phase shift of 73° (Supplementary Fig. 7). No phase-shift-based filtering was applied, ensuring that the alignment comparison reflects representative conditions from routine VPP cryo-ET data collection.

Comparing alignments between VPP tomograms of the two-protein phantom and the minicell datasets, improved alignments were noted for the more crowded and thicker tomograms of the minicell. The measured tilt-axis orientation in the VPP dataset clustered more tightly around the calibrated instrument axis, exhibiting an ∼2.5-fold lower standard deviation (9.18° versus 23.07°) and a median value closer to the calibrated orientation (−95.98° for VPP versus −96.39° for non-VPP, relative to −96.06°) [Fig. 4 ▸(*c*)]. Local alignment quality also improved: 61% of VPP tomograms showed no problematic local shifts, compared with 42% for the non-VPP dataset [Fig. 4 ▸(*d*)]. Table 4 ▸ summarizes the statistics for the minicell tomogram alignment comparison. For the thinner and less-crowded two-protein phantom datasets, VPP and non-VPP tomograms exhibit similarly high alignment quality (Supplementary Fig. 8), indicating the VPP primarily benefits datasets that are more challenging to align.

Tomogram alignment becomes more challenging for thicker and denser specimens. In addition to acquiring the data from the same sample-preparation batch, we filtered the datasets to match the minimum and maximum post-specimen dose ranges between conditions (see Section 2.8). After filtering, we compared sample thickness measured independently by *AreTomo3* [Fig. 4 ▸(*e*)]. The measured thickness distributions show similar mean and median values. The larger standard deviation in the non-VPP dataset is likely due to increased uncertainty in thickness estimation, which results from poorer tomogram alignment as the thickness measurement depends on alignment quality. Together, these controls indicate that the observed improvements in alignment quality are not driven by systematic differences in specimen thickness. We also note that although non-VPP tomograms were acquired using parallel acquisition (multiple tilt series at a stage position) whereas VPP tomograms were acquired without parallel acquisition, excluding image-shifted non-VPP tomograms did not alter these conclusions (Supplementary Fig. 9).

### 3.5. Subtomogram averaging with and without VPP

**Figure 5:**
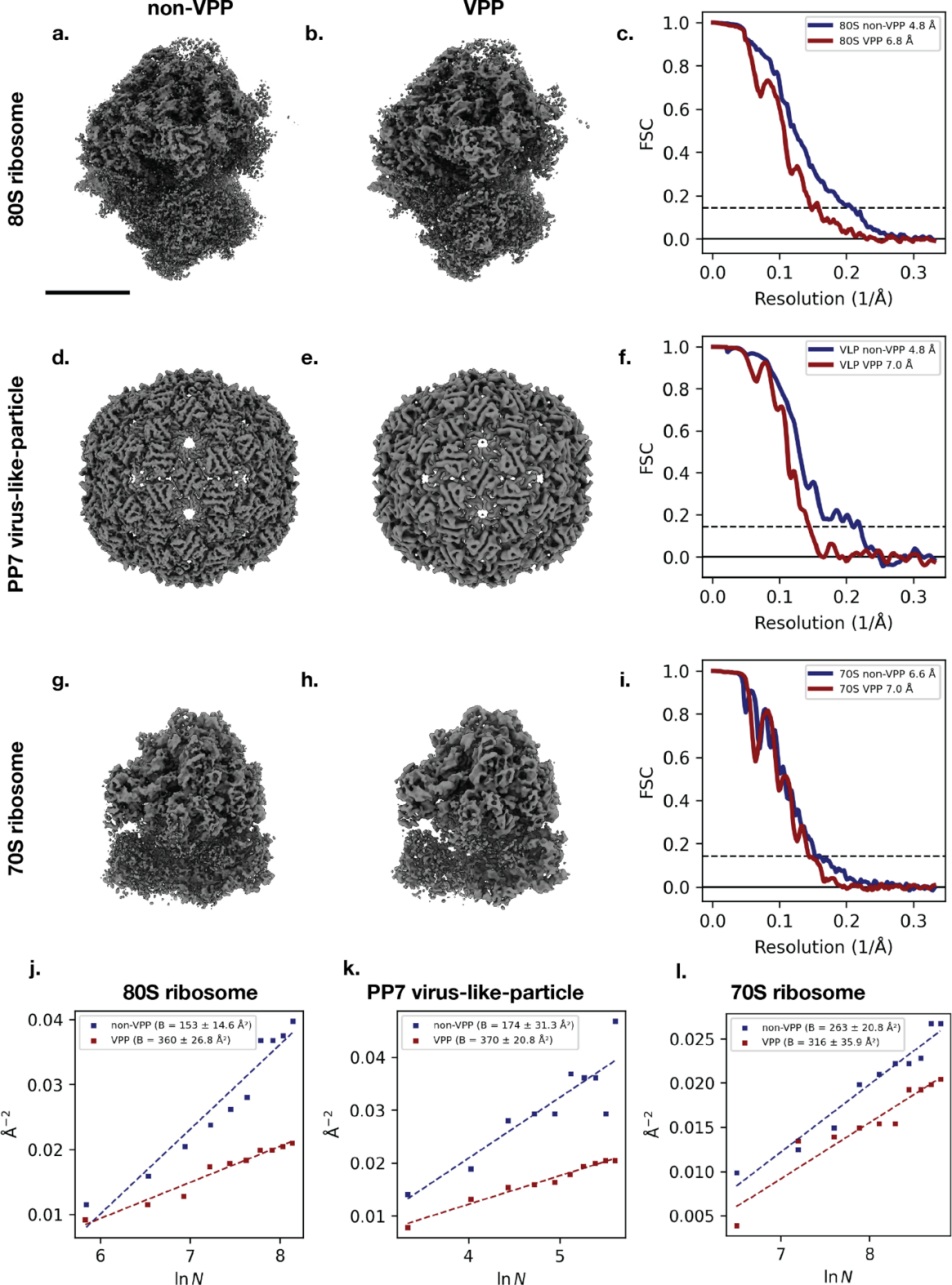
Subtomogram average of 80S ribosomes and PP7 virus-like particles (VLPs) from two-protein phantom, and 70S ribosomes from minicells, with and without the Volta phase plate (VPP). (*a*, *b*) Subtomogram averaging of the 80S ribosome obtained from non-VPP (*a*) and VPP (*b*) tomograms. (*c*) Fourier shell correlation (FSC) curves for the 80S ribosome, indicating a resolution of 4.8 Å for the non-VPP dataset and 6.8 Å for the VPP dataset using the FSC = 0.143 criterion. (*d*, *e*) Subtomogram averaging of PP7 virus-like particles (VLPs) from non-VPP (*d*) and VPP (*e*) tomograms. (*f*) FSC curves for the PP7 VLP reconstructions, indicating a resolution of 4.8 Å from the non-VPP datasets and 7.0 Å from the VPP datasets. (*g*, *h*) Subtomogram averaging of 70S ribosomes from non-VPP (*g*) and VPP (*h*) tomograms. (*i*) FSC curves for the 70S ribosome reconstructions, indicating a resolution of 6.6 Å from the non-VPP datasets and 7.0 Å from the VPP datasets. (*j*–*l*) Rosenthal–Henderson *B*-factor plots for non-VPP (blue) and VPP (red) datasets for 80S ribosomes, VLPs and 70S ribosomes, as indicated. Scale bars: 10 nm. Maps are displayed as isosurfaces at 6, 5 and 6 standard deviations from the mean for the 80S ribosome, VLP and 70S ribosome, respectively.

To assess how the VPP affects subtomogram averaging (STA), we first compared 80S ribosomes volumes obtained from two-protein phantom tomograms acquired with VPP and without VPP [Fig. 5 ▸(*a*)–(*c*)]. The same STA processing pipeline was applied, and the same number of particles (Table 2 ▸) was used. The non-VPP map reached 4.8 Å, compared with the VPP map at 6.8 Å, based on the FSC = 0.143 criterion, with *B* factors of 153 Å^2^ and 360 Å^2^, respectively [Fig. 5 ▸(*j*)]. Despite this difference, both reconstructions resolve secondary-structure elements, demonstrating that the VPP dataset is still capable of structure determination of large macromolecular assemblies.

For PP7 virus-like particles (VLPs), which were processed using the same workflow and matched particle numbers [Table 2 ▸; Fig. 5 ▸(*d*)–(*f*)], the non-VPP reconstruction again reached 4.8 Å, compared with the VPP reconstruction at 7.0 Å, with *B* factors of 174 Å^2^ and 370 Å^2^, respectively [Fig. 5 ▸(*k*)]. As with the ribosomes, both maps reveal recognizable secondary-structure features, while the non-VPP map retains higher-resolution features.

For 70S ribosomes from minicells, we observe higher resolution for the non-VPP reconstructions but to a lesser extent, reaching 6.6 Å and 7 Å for non-VPP and VPP datasets, respectively [Fig. 5 ▸(*g*)–(*i*)]. This is reflected by more-similar *B* factors of 263 Å^2^ and 316 Å^2^, respectively [Fig. 5 ▸(*l*)], the potential reasons for which are discussed below.

To assess the benefit of each refinement step for VPP and non-VPP STA, we performed an ablation test in our STA pipeline where CTF refinement or Bayesian polishing steps were omitted (Supplementary Fig. 11). All non-VPP datasets demonstrate the combined benefits of CTF refinement and Bayesian polishing [Supplementary Fig. 11(*a*, *c*, *e*)], consistent with previously reported trends with similar pipelines (Zivanov *et al.*, 2022 ▸; Zivanov *et al.*, 2020 ▸). However, for VPP datasets, the full STA pipeline demonstrates a similar resolution and map quality to an STA pipeline with CTF refinement omitted [Supplementary Fig. 11(*b*, *d*, *f*)]. This suggests that in our hands, current CTF refinement capabilities for VPP data require further testing for potential resolution improvements – for example, whether including phase-shift refinement helps. However, the low defocus values of the VPP dataset and the VPP-introduced signal loss may also be the limiting factors. We note that in the case of 70S ribosomes from minicells prior to CTF refinement and Bayesian polishing, the VPP dataset obtains a better resolution than non-VPP with the same number of particles, reaching 8.2 Å and 9.5 Å, respectively. This could suggest that the VPP provides a benefit for STA in intermediate resolution ranges for more crowded specimens. A systemic diagnosis of these sample types and data processing strategies will be important to explore the limit of VPP STA resolution.

## 4. Discussion

By combining single-particle benchmarks, analytical contrast transfer function (CTF) modeling and quantitative alignment metrics, we demonstrated that the VPP is expected to enhance low- to medium-frequency signals despite the attenuation of high-frequency information. This enhanced mid- to low-frequency signal translates into better tilt-series alignment, particularly for thicker and more crowded specimens where alignment is usually challenging.

Our sub-2 Å single-particle analysis of apoferritin provided an empirical measure of VPP-induced attenuation at high spatial frequencies. When this measured attenuation was incorporated into analytical contrast transfer function (CTF) calculations, the model predicted enhanced signal in the frequency range most relevant for motion correction and tilt-series alignment. It further suggests that the largest signal gains would be achieved at close-to-zero defocus combined with phase shifts at 90°. In practice, however, robust CTF estimation and correction at near-zero defocus remains challenging, and achieving stable ∼90° phase shifts with the VPP is operationally difficult and associated with low throughput. As next-generation phase-plate technologies such as the laser phase plate (Schwartz *et al.*, 2019 ▸) mature, it will become increasingly important to develop advanced CTF estimation and correction methods tailored for close-to-zero defocus data.

Experimentally, we observed that VPP-aligned tilt series showed tighter clustering of measured tilt-axis orientations and fewer problematic local alignments compared with non-VPP data. In contrast, for a relatively thin and sparsely packed two-protein phantom sample, we did not see a noticeable alignment improvement from the VPP, suggesting the VPP’s benefits become most evident for difficult samples. In this work we primarily evaluated alignment quality using *AreTomo3*’s metrics; an important next step will be to assess how these alignment improvements translate to interpretation and downstream processing. For example, future studies could quantify alignment gains by assessing single-tomogram resolution or template matching performance prior to subtomogram averaging.

Our STA comparison also indicated that the VPP sub­tomogram averages were lower resolution than non-VPP, consistent with previous reports (Khoshouei *et al.*, 2017 ▸; Turoňová, 2020 ▸), but still resolved 80S ribosomes and virus-like-particles at ∼7 Å. This inferior STA performance with the VPP is partly due to the damping of high-frequency signals by the VPP and potentially compounded by the lack of phase-shift-aware refinement in our STA workflow. We anticipate that phase-shift-aware refinement available in other pipelines (Tegunov *et al.*, 2021 ▸) could help improve the resolution for VPP datasets. Nevertheless, focusing solely on STA resolution might overlook practical advantages like improved contrast and tilt-series alignment that the VPP confers earlier in the processing pipeline.

In most microscope optical designs, large image shifts can displace the unscattered beam from the VPP phase-shift patch; therefore, parallel image-shift-based acquisition was not used in this study, resulting in a nearly order-of-magnitude reduction in data-collection throughput. In addition, the VPP requires precise on-plane alignment at the microscope back focal plane, and electrostatic charging, particularly prevalent in focused ion beam (FIB)-milled lamellae, can perturb this alignment and degrade phase-plate performance. Future improvements in microscope column design that enable image shifting without altering phase-plate alignment, together with strategies to mitigate specimen charging, will be critical for scaling up throughput for phase-plate-based cryo-ET and extending to lamella workflows. Addressing these limitations would substantially improve the throughput and scope of phase-plate tomography and benefit both the VPP and next-generation phase plates, including laser phase plates.

Taken together, our findings demonstrate that despite high-frequency signal attenuation, the VPP provides a benefit for cryo-ET by improving tilt-series alignment in challenging specimens. Enhanced alignment robustness is expected to propagate into more reliable and interpretable tomographic reconstructions. By improving phase-plate imaging throughput and data processing, future studies can leverage both the VPP and emerging next-generation phase plates to push the limits of cryo-ET structural analysis.

## Supplementary Material

Supplementary figures 1-11. DOI: 10.1107/S2052252526002575/rq5018sup1.pdf

EMDB reference: PP7 virus-like-particle without VPP (full dataset), EMD-75881

EMDB reference: PP7 virus-like-particle with VPP (full dataset), EMD-75882

EMDB reference: PP7 virus-like-particle with VPP (partial dataset), EMD-75890

EMDB reference: 80S ribosome without VPP (full dataset), EMD-75895

EMDB reference: 80S ribosome without VPP (partial dataset), EMD-75896

EMDB reference: 80S ribosome with VPP (full dataset), EMD-75897

EMDB reference: 70S ribosome without VPP (full dataset), EMD-75898

EMDB reference: 70S ribosome without VPP (partial dataset), EMD-75899

EMDB reference: 70S ribosome with VPP (full dataset), EMD-75900

EMDB reference: apoferritin without VPP, EMD-75265

EMDB reference: apoferritin with VPP, EMD-75263

Tomograms DS-10452, DS-10442, DS-10469 and DS-10468 (VPP minicell, non-VPP minicell, and two-protein phantom with VPP and without VPP, respectively): https://cryoetdataportal.czscience.com

## Figures and Tables

**Table 1:** Particle picking of the VLP and the 80S ribosome structures from the two-protein phantom with and without the VPP

	VLPs		80S ribosome	
	Non-VPP	VPP	Non-VPP	VPP
Total tomograms	197	115	197	115
Manual picks	22	25	225	159
Tomograms with manual picks	14	1	4	3
*Octopi* picks	490	514	15643	8996
Post 2D minislab classification picks	290	323	11479	3710
Post 2D minislab retain rates (%)	59.1	62.8	73.4	41.2

**Table 2:** Data collection and processing for cryo-ET STA of the VLP and the 80S ribosome structures from the two-protein phantom, and the 70S ribosomes from minicells, with and without the VPP Particle counts and resolutions are reported for full datasets, in addition to the equalized datasets for VPP versus non-VPP comparison. VLP symmetry: *I*. 80S and 70S ribosome symmetry: *C*_1_. All datasets: voltage 300 kV; tilt series (min/max, increment) +/−45°, 3°; total electron exposure 120 e A^−2^; pixel size 1.51 Å pixel^−1^.

	VLP			80S ribosome			70S ribosome		
	Non-VPP	VPP	VPP (full)	Non-VPP	Non-VPP (full)	VPP	Non-VPP	Non-VPP (full)	VPP
Nominal defocus range (µm)	2	0.7–0.9		2		0.7–0.9	2		0.7–0.9
Particles	271	270	301	3436	10406	3408	6635	14258	6636
Map resolution (FSC 0.143 Å)	4.8	7.0	6.8	4.8	3.9	6.8	6.6	4.8	7.0
EMDB ID	EMD-75881	EMD-75890	EMD-75882	EMD-75896	EMD-75895	EMD-75897	EMD-75899	EMD-75898	EMD-75900

**Table 3:** Data collection and processing summary for apoferritin SPA with and without the VPP

	non-VPP	VPP
Median defocus (µm)	0.95	0.63
Micrographs with CTF fit < 4 Å	74%	82%
Targeted phase shift	—	∼60°
Micrographs with phase shift > 30°	—	54%
Particles with 2D classification < 4 Å	73%	38%
Particles with 2D classification < 4 Å using HPF	74%	71%
Particles used for reconstruction	300 702	300 702
FSC map resolution (Å)	1.66	1.90
Rosenthal–Henderson *B* factor (Å^2^)	67.6	100.8
EMDB ID	EMD-75265	EMD-75263

**Table 4:** Summary of tomogram alignment statistics for VPP and non-VPP minicell datasets Std Dev = standard deviation.

	VPP	non-VPP
Total tomograms	110	319
Filtered by post-specimen dose	110	305
Tilt-axis orientation median	−95.98°	−96.39°
Tilt-axis orientation Std Dev	9.18°	23.07°
Zero problematic local shift	61%	42%
Problematic local shift (mean)	4%	8.5%
Sample thickness (mean, median, Std Dev) (Å)	2627, 2648, 536	2492, 2602, 831

## Data Availability

The VPP minicell tomogram is available through the CryoET Data Portal (https://cryoetdataportal.czscience.com) under accession DS-10452. The non-VPP minicell dataset acquired is available with ID DS-10442. Tomograms and annotations of the two-protein phantom sample acquired with and without the VPP are under accessions DS-10469 and DS-10468, respectively. All maps have been deposited with the EMDB with accession codes summarized in Tables 2 ▸ and 3 ▸. *Octopi* (https://github.com/chanzuckerberg/octopi) was used for particle picking. *Slabpick* (https://github.com/apeck12/slabpick) and *py2rely* (https://github.com/chanzuckerberg/py2rely/tree/kaggle) were used for minislab-based 2D classification and curation, and *py2rely* was used for cryo-ET subtomogram averaging.
