# Supplementary material for: Evaluating the Volta phase plate for improved tomogram alignment in cryo-electron tomography
Source: IUCrJ. 2026 Apr 9;13(Pt 3):260–72. doi: 10.1107/S2052252526002575 (PMC13134490; doi:10.1107/S2052252526002575)
Supplement: Supplementary file 1 [file m-13-00260-sup1.pdf]

IUCrJ

Volume 13 (2026)

Supporting information for article:

Evaluating the Volta phase plate for improved tomogram alignment in cryo-electron tomography

Joshua Hutchings, Daniel Ji, Shawn Zheng, Elizabeth A. Montabana, Utz H. Ermel, Ariana Peck, Jonathan Schwartz, Rahel Woldeyes, Mohammadreza Paraan, Mallak Ali, Norbert S. Hill, Hannah Siems, Daniel Serwas, Anchi Cheng, Dari Kimanius, David A. Agard, Clinton S. Potter, Bridget Carragher and Yue Yu

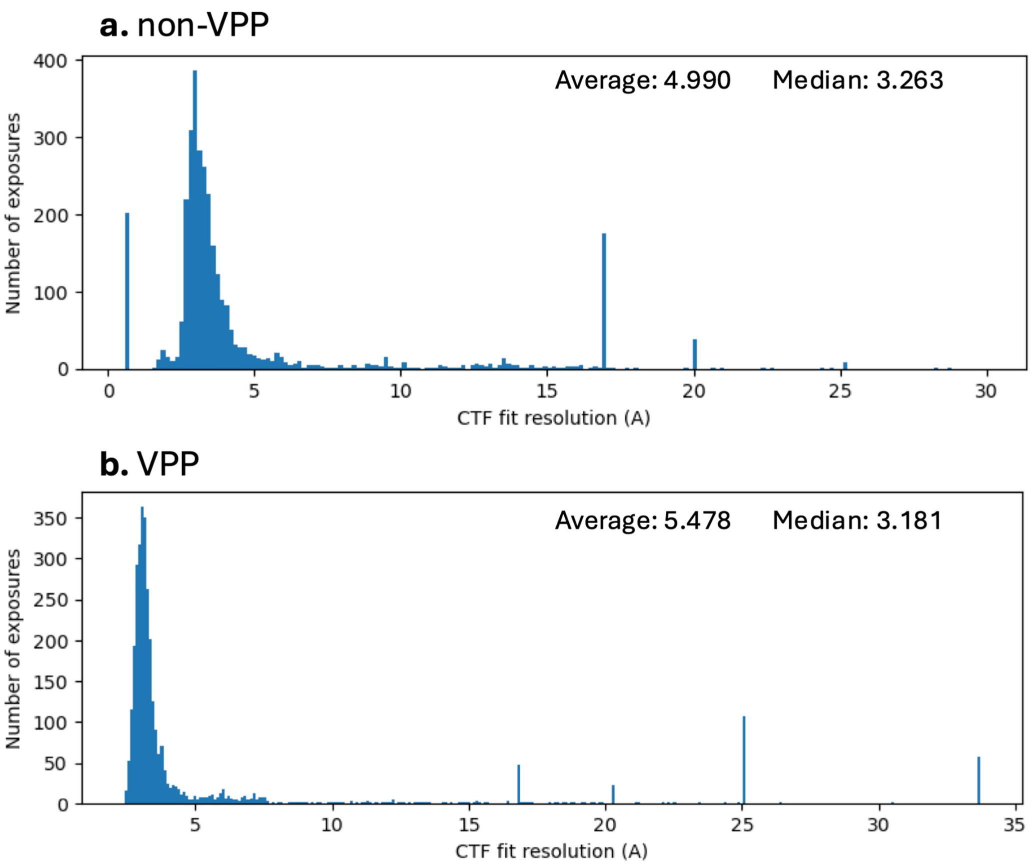

Supplementary Figure 1 | cryoSPARC patch CTF fit resolution for non-VPP and VPP datasets. **a)** Distribution of CTF fit resolution for the non-VPP dataset (average 4.99 Å, median 3.26 Å). **b)** Distribution of CTF fit resolution for the VPP dataset (average 5.48 Å, median 3.18 Å). Overall, the average CTF fit resolution of the VPP dataset is ~9.8% worse than that of the non-VPP dataset, but the median CTF fit resolution is ~2.5% better for the VPP dataset.

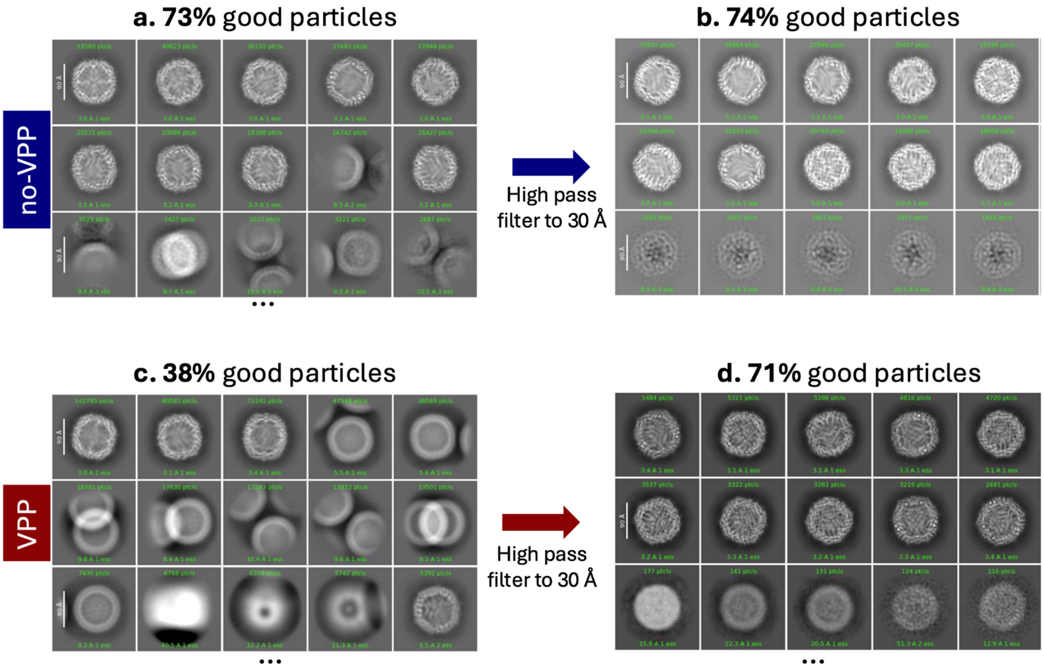

Supplementary Figure 2 | Representative 2D class averages from non-VPP and VPP datasets with and without high-pass filtering.

a,b) Non-VPP dataset without (a) and with (b) high-pass filtering at 30 Å during 2D classification. With high-pass filtering, spatial frequencies lower than 30 Å were not used. High-pass filtering has a marginal effect on the fraction of particles in good classes (73% without filtering and 74% with filtering). **c,d)** VPP dataset without (c) and with (d) high-pass filtering at 30 Å during 2D classification. In contrast to non-VPP, high-pass filtering substantially improves the fraction of particles in good classes for the VPP dataset, increasing from 38% to 71%. Good particles are defined as those belonging to 2D classes with resolution better than 4 Å.

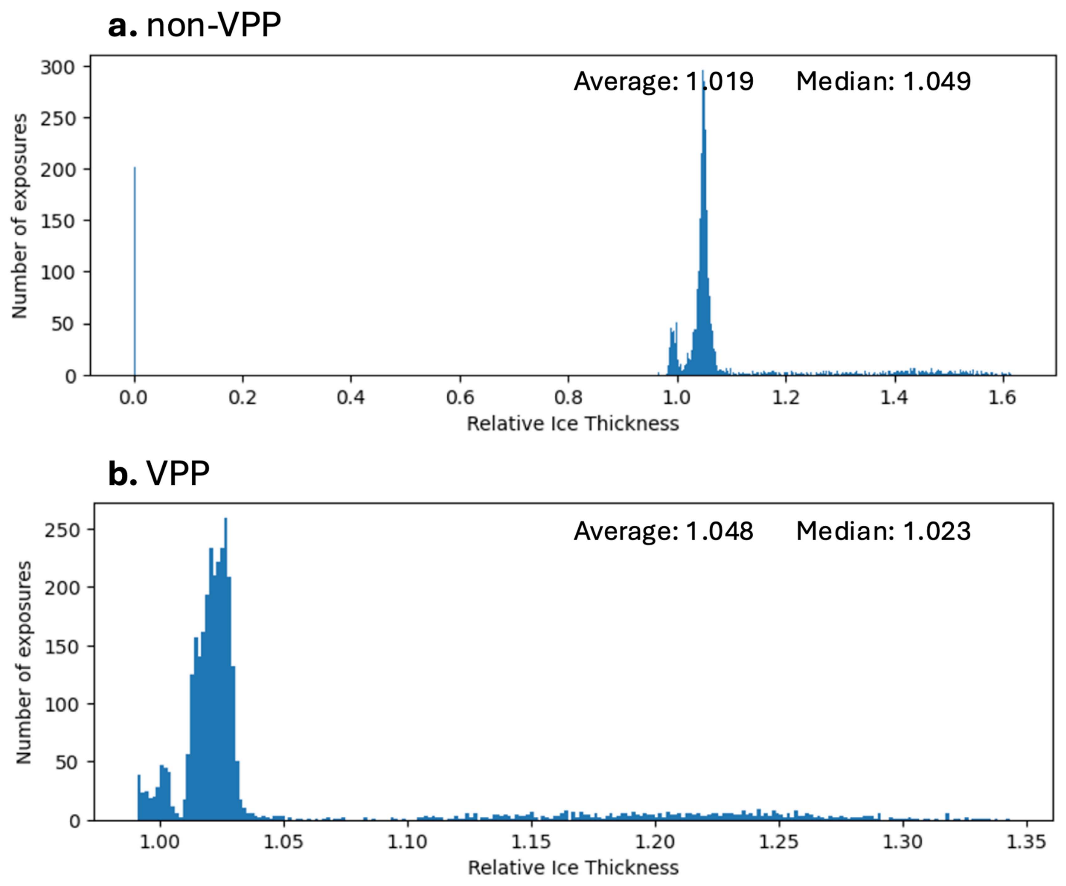

Supplementary Figure 3 | Relative ice thickness estimated by cryoSPARC[30] for non-VPP and VPP datasets.

a) Distribution of relative ice thickness for the non-VPP dataset (average 1.019, median 1.049). **b)** Distribution of relative ice thickness for the VPP dataset (average 1.048, median 1.023). Relative ice thickness was estimated by cryoSPARC from CTF fits. Overall, the two datasets show comparable ice thickness distributions, with similar average and median values.

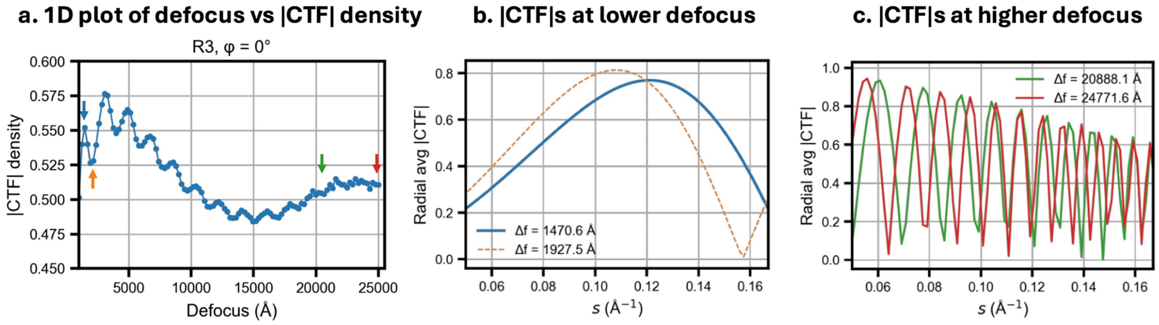

Supplementary Figure 4 | Defocus-dependent oscillation of CTF density in the R3 spatial-frequency band. a) One-dimensional defocus sweep of the integrated CTF density in the R3 band (0.05–0.167 $Å^{-1}$) at phase shift φ = 0°. The curve exhibits multiple local extrema as a function of defocus (blue and orange arrows). **b)** |CTF| profiles, shown for 2 representative lower defocus values (Δf ≈ 1471 Å and 1928 Å). Solid lines indicate defocus values corresponding to a local maximum of the |CTF| density, and dashed lines indicate local minima. **c)** |CTF| profiles at higher defocus values. At these defocus values, the more rapid CTF oscillations across spatial frequency reduce the prominence of distinct local maxima in the integrated CTF density as a function of defocus.

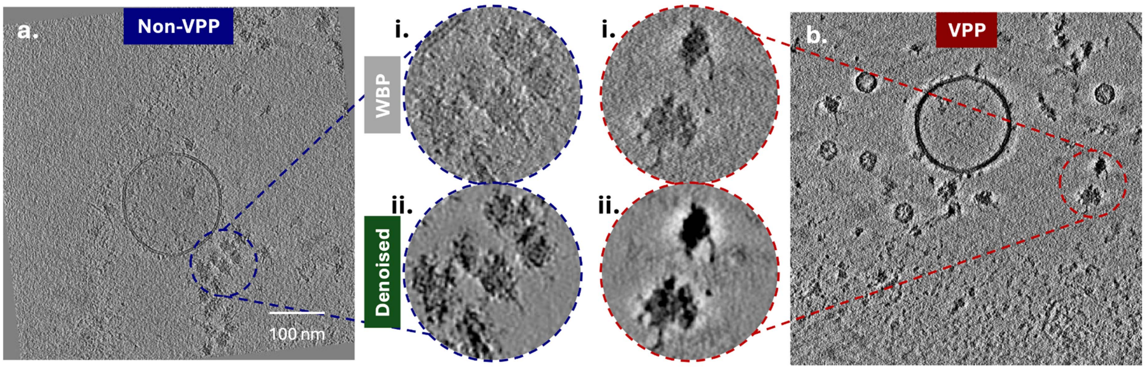

Supplementary Figure 5 | Visualization of two-protein phantom tomograms acquired with and without a Volta phase plate (VPP)

a–b) 2-nm slabs from the two-protein phantom recorded without VPP (a; 140 nm thickness measured by Aretomo3, 2.4 µm defocus) and with VPP (b; same as in Fig. 3b). Zoom-in regions highlight selected areas reconstructed using weighted back-projection (i) and after denoising (ii).

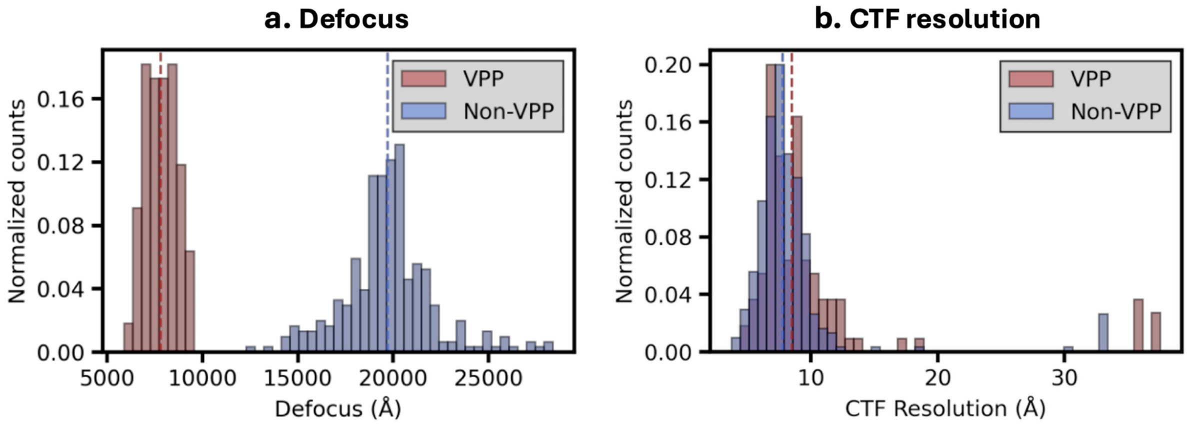

Supplementary Figure 6 | Defocus and CTF fit quality for VPP and non-VPP minicell cryo-ET datasets.

a) Distribution of measured defocus values for VPP and non-VPP tomograms. The measured median defocus is 0.78 µm for the VPP dataset and 1.97 µm for the non-VPP dataset. **b)** Distribution of estimated CTF resolution for VPP and non-VPP tomograms. The median CTF resolution is 8.48 Å for the VPP dataset and 7.7 Å for the non-VPP dataset. Dashed lines indicate median values.

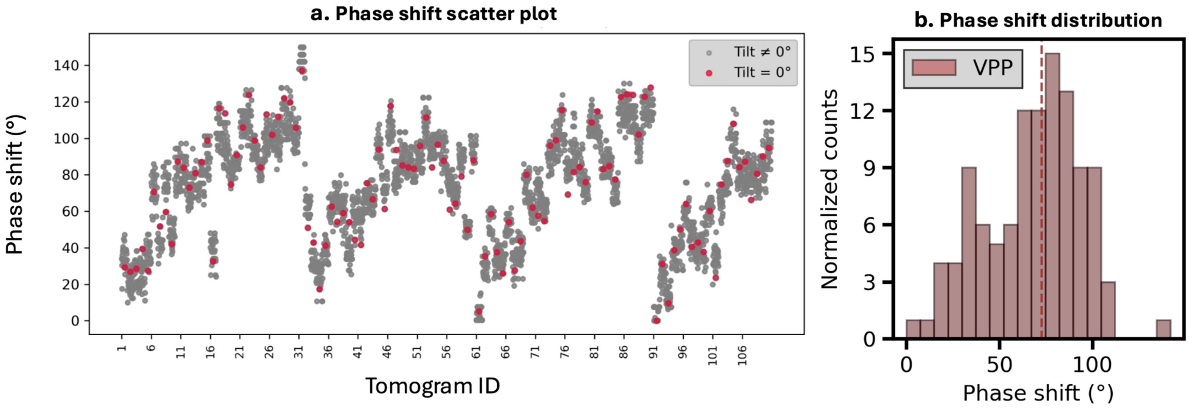

Supplementary Figure 7 | Phase-shift evolution and distribution for VPP cryo-ET datasets.

a) Scatter plot of measured phase shifts for individual tilt images plotted in acquisition order. Phase shifts evolve over time acquisition as expected; gray points indicate tilted images (tilt ≠ 0°) and red points indicate the first 0-tilt images. **b)** Distribution of phase shifts across all VPP tomograms. The median phase shift is 73°. Dashed lines indicate the median value.

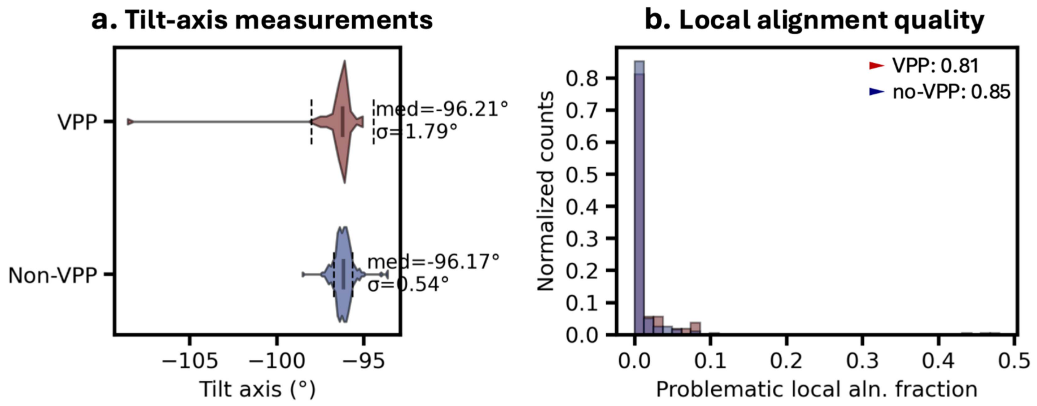

Supplementary Figure 8 | Tomogram alignment quality for VPP and non-VPP cryo-ET datasets of the 2-protein phantom, relatively thin compared to the minicells (mean thickness 1150 Å for VPP and 1227 Å for non-VPP).

a) Distributions of measured tilt-axis orientations for VPP and non-VPP tomograms of the two-protein phantom dataset. Both datasets show tightly clustered tilt-axis measurements, with non-VPP's Std Dev slightly smaller reflecting with comparable medians (−96.21° for VPP and −96.17° for non-VPP). **b)** Distributions of problematic local alignment fractions reported by Aretomo3. For this thinner and sparsely populated sample, both VPP and non-VPP datasets exhibit similarly high fractions of tomograms with zero problematic local shifts (81% for VPP and 85% for non-VPP).

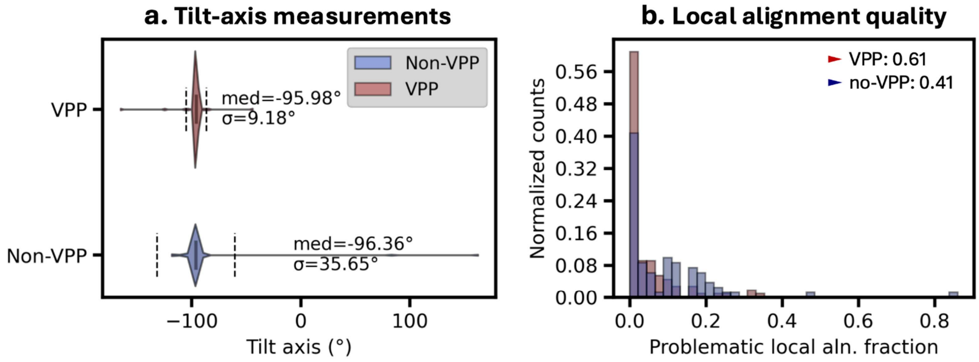

Supplementary Figure 9 | Excluding image-shifted parallel acquisitions of non-VPP tomograms, alignment quality for VPP and non-VPP similar to Figure 4c) and d).

a) Distributions of measured tilt-axis orientations for VPP and non-VPP tomograms. The VPP dataset shows a tighter distribution. **b)** Distributions of problematic local alignment fractions reported by Aretomo3.

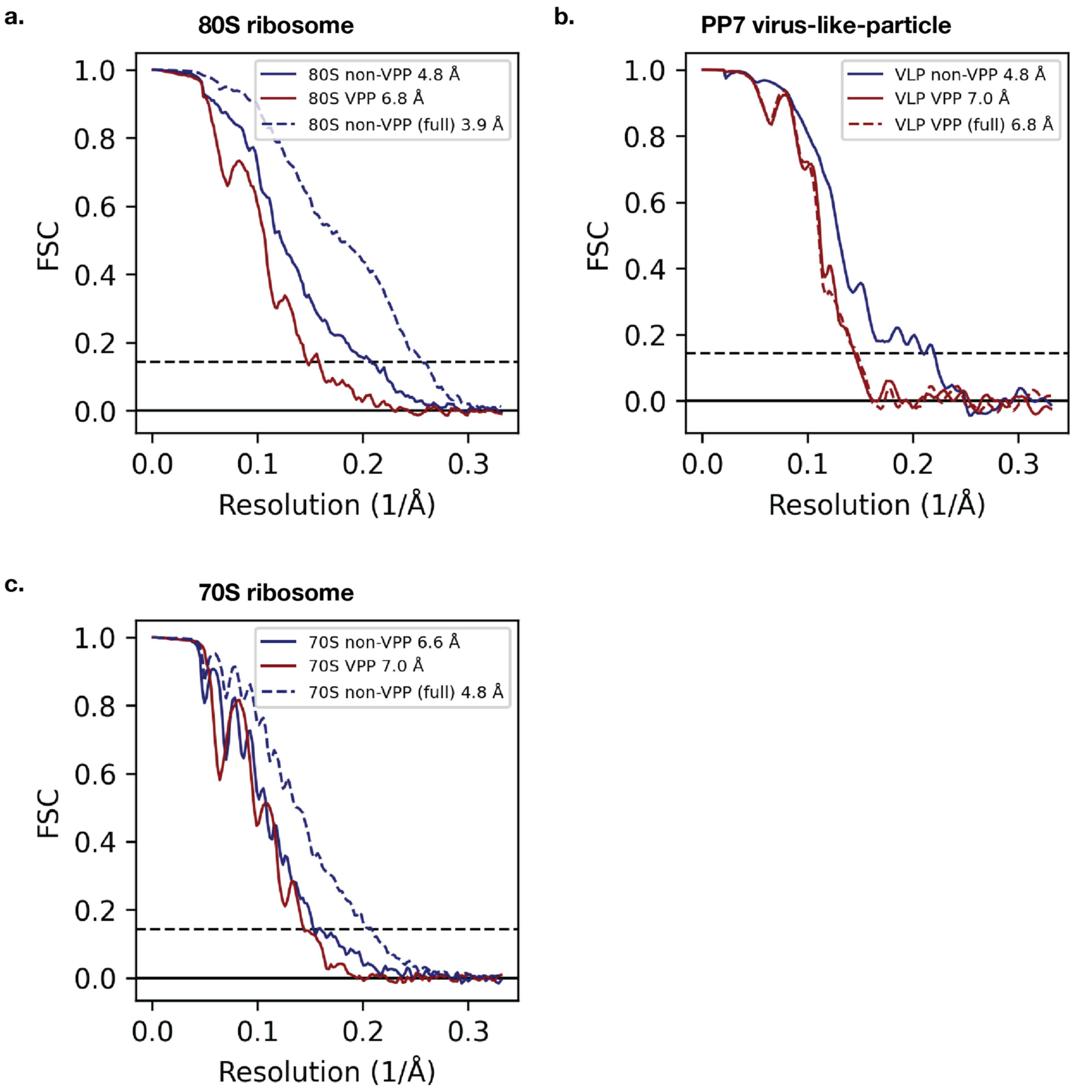

Supplementary Figure 10 | Half-map FSCs for full PP7 virus-like-particle and 80S ribosome, PP7 virus-like-particle and 70S ribosome datasets.

a-c) The same half-map FSCs from Figure 5 are plotted with the addition of the full dataset half-map FSCs (dashed lines), from which the random subset was obtained for equal particles. In the case of 80S and 70S ribosomes, the non-VPP dataset had more particles (blue). In the case of VLPs, the VPP dataset had more particles (red).

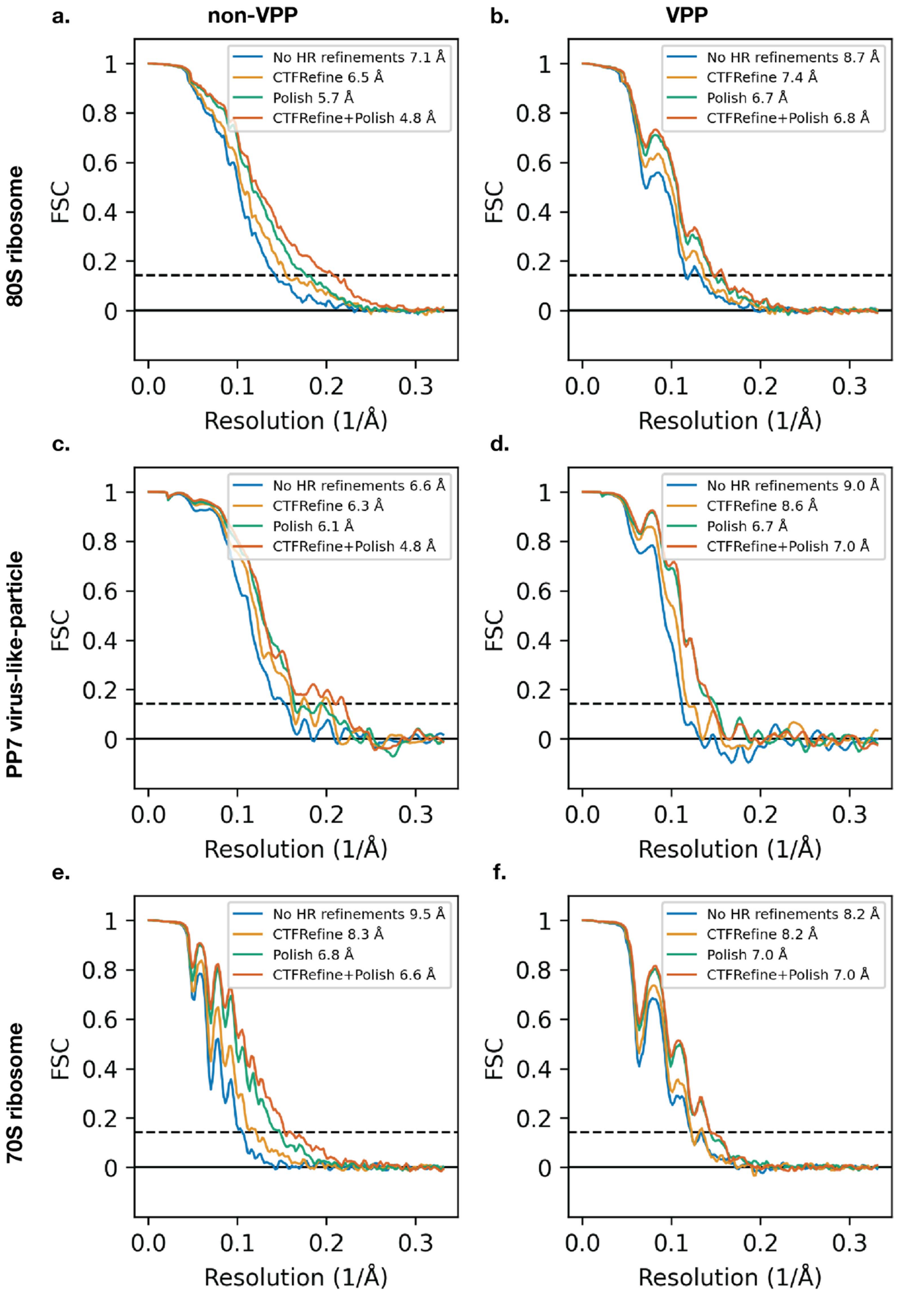

Supplementary Figure 11 | Ablation test for subtomogram averaging pipeline of non-VPP and VPP datasets.

a-f) Half-map FSCs for maps without higher-resolution (HR) refinements (blue), CTF refinement only (“CTFRefine”, yellow), Bayesian polishing only (“Polish”, green), and the full pipeline with CTF refinement and polishing (“CTFRefine+Polish”, red).
